# The Lake Erie Dental Association

**Published:** 1867-11

**Authors:** 


### THE LAKE ERIE DENTAL ASSOCIATION.

This Body held its regular meeting in September last, at Westfield, N. Y. This society is in active working operation, composed of good men and true, who have the best interest of their chosen profession at heart; and are doing all they can in associated capacity for its support and development. The papers read were good, and evinced much thought and research. The discussions were warm, earnest, and interesting; having for their aim the unfoldment of the truth, and its application for the progress of the science and art of our profession.

This association will work great good for the profession in that region of the country.

The subject of legal recognition and protection was discussed, and a committee was appointed to take measures to secure the desired legislation. We feel confident that this society will perform its full measure of work, for its members are wide awake. T.

